# PEG-embedded KBr_3_: A recyclable catalyst for multicomponent coupling reaction for the efficient synthesis of functionalized piperidines

**DOI:** 10.3762/bjoc.7.157

**Published:** 2011-09-28

**Authors:** Sanny Verma, Suman L Jain, Bir Sain

**Affiliations:** 1 Chemical Sciences Division, Indian Institute of Petroleum (Council of Scientific and Industrial Research), Dehradun-248005, India

**Keywords:** heterocycles, metal free synthesis, multicomponent coupling, piperidine, recyclable catalyst

## Abstract

PEG-embedded potassium tribromide ([K^+^PEG]Br_3_^−^) was found to be an efficient and recyclable catalyst for the synthesis of functionalized piperidines in high yields in a one step, three component coupling between aldehyde, amine and β-keto ester. At the end of the reaction the [K^+^PEG]Br_3_^−^ was readily regenerated from the reaction mixture by treating the residue containing [K^+^PEG]Br^−^ with molecular bromine.

## Introduction

Multicomponent reactions [1–5], involving the one-pot reaction of three or more components to produce valuable compounds, have been recognized as one of the important tools to achieve highly efficient, atom economic and energy-saving organic syntheses. These multicomponent reactions (MCRs) offer many advantages over conventional multistep syntheses, such as lower costs, shorter reaction times, high atom economy, avoidance of expensive purification processes [6–7], and the fact that it is more environmentally friendly [4–5,8–9]. Among the various known multicomponent reactions, the MCRs that involve 1,3-dicarbonyl compounds, aldehydes, and nucleophilic compounds have received particular interest in recent years owing to their potential to provide different condensation products depending on the specific conditions and structures of the building blocks. The synthesis of highly functionalized piperidines is an important synthetic transformation [10–14] as these compounds find extensive applications in the synthesis of a number of organic fine and bioactive compounds [15–17]. Also the piperidine ring is present in many natural products [18–19] such as alkaloids, which are responsible for a number of unique activities including anti-hypertensive [20], anticonvulsant and anti-inflammatory activities [21]. Many conventional methods, such as imino Diels–Alder reactions [22–23], intramolecular Michael reactions [24], intramolecular Mannich reactions onto iminium ions [25], tandem cyclopropane ring-opening/Conia-ene cyclizations [26], and aza-Prins-cyclization [27–29], have been reported for the synthesis of piperidines. However, these methods suffer from the drawbacks of multistep synthesis and a lower yield of the desired products. On the other hand, the one-step coupling between an aldehyde, an amine and a 1,3-dicarbonyl compound represents a straightforward approach for their synthesis. Recently, a variety of improved methods employing tetrabutylammonium tribromide (TBATB) [30], bromodimethylsulfonium bromide (BDMS) [31], InCl_3_ [32] and L-proline/TFA [33] were reported for the one-pot syntheses of these heterocyclic compounds. However, tedious synthetic procedures, the need for high catalyst loadings, and the expense and non-recyclability of the catalyst make these methods of limited utility. Thus, the development of a simple, cost-effective and recyclable catalyst for the efficient preparation of functionalized piperidines is particularly desired.

## Results and Discussion

**Scheme 1:**
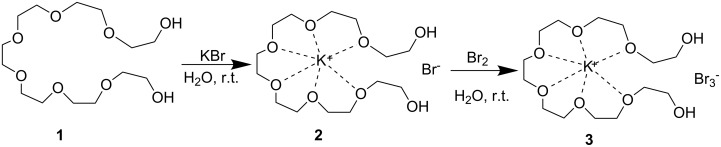
Synthesis of [K^+^PEG]Br_3_^−^.

In the present paper, we report a simple, highly efficient and easily accessible catalytic protocol for the synthesis of functionalized piperidines by the one-pot coupling of aldehyde, amine and β-keto ester with PEG-wrapped KBr_3_ as a recyclable catalyst. The PEG-embedded tribromide could easily be synthesized through the concept of host–guest complexation [34] of PEGs with alkali metal cations, as shown in Scheme 1. The developed catalyst **3** was readily prepared by the mixing of equimolar amounts of PEG_400_ (**1**) and KBr to give [K^+^PEG]Br^−^ **2**, which on subsequent reaction with Br_2_ resulted in a dark orange-red colored viscous liquid (see Experimental). The liquid was dried under vacuum and used as such for the present synthesis. Poly(ethylene)glycols are well known to have similar structures to crown ethers and therefore we were able to assume the structure of the [K^+^PEG]Br_3_^−^ as being similar to [18-crown-6]KBr_3_ [35] (Scheme 1).

**Scheme 2:**
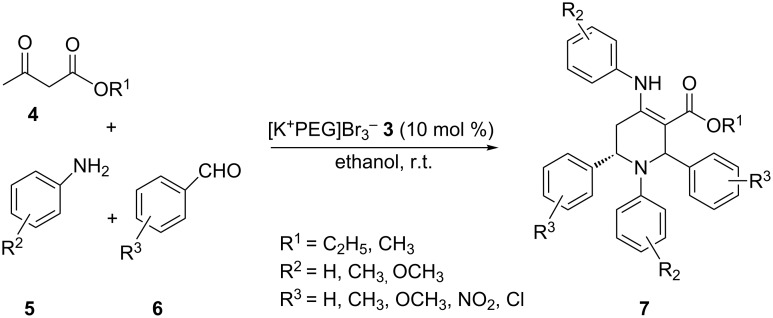
Synthesis of functionalized piperidines.

The protocol developed for the synthesis of piperidines **7** involves the one-step reaction between β-keto ester **4**, aniline **5**, aldehyde **6** in the molar ratio 2:2:1 with **3** (10 mol %) as catalyst in ethanol at room temperature (Scheme 2).

**Figure 1:**
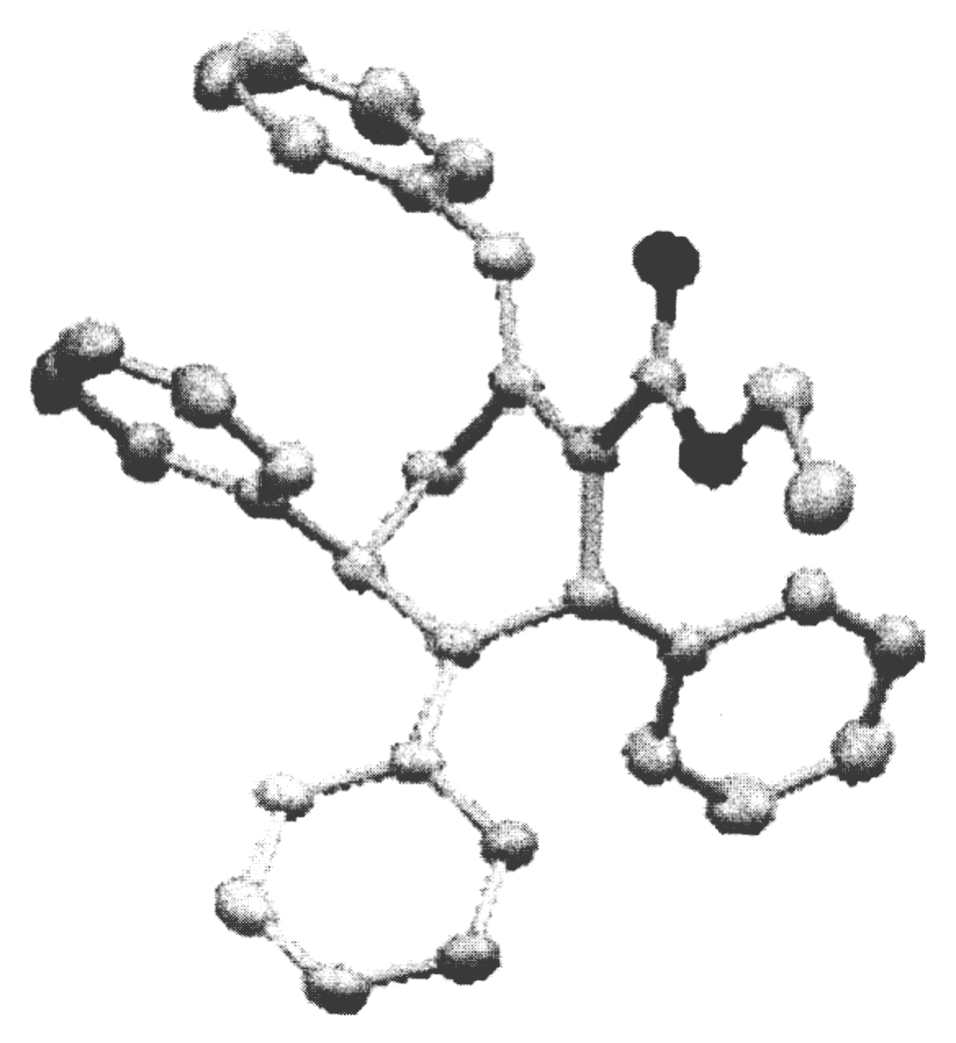
X-ray structure showing the anti orientation of the phenyl rings at C2 and C6.

At first we studied the coupling between benzaldehyde, aniline and ethyl acetoacetate under the described reaction conditions (see Experimental). The reaction proceeded efficiently and afforded a high product yield under mild reaction conditions. These successful results prompted us to extend the scope of the reaction with a variety of aldehydes, substituted anilines and β-keto esters under identical reaction conditions. The results of these experiments are summarized in Table 1. In all cases we achieved moderate to high yields of the products, which were identified by comparing their physical and spectral properties with those of authentic samples. Among the various anilines, the reaction was found to be faster with anilines having electron donating groups, whereas the reaction was found to be slow with aniline, which contains an electron-withdrawing group (Table 1, entry 12). Similarly the reaction was found to be very slow in the case when both aniline and aldehyde were substituted with electron-withdrawing groups and gave only a trace amount of the product (Table 1, entry 13). The combination of benzaldehyde, aniline, and a β-keto ester having a *tert*-butyl group in the β-position, led to the formation of Mannich-type product instead of the desired piperidine **7** (Table 1, entry 14). The use of other organic solvents such as acetonitrile, dichloromethane and toluene affected the reaction adversely and we achieved a poor yield of the corresponding coupling product. Similarly, at high reaction temperature the selectivity of the product was decreased, and an intricate mixture of unidentified products was generated. The relative stereochemistry at the C2 and C6 position was found to be anti, as confirmed by X-ray crystallographic analysis of **7a** (Figure 1).

**Table 1:** Synthesis of functionalized piperidines with [K^+^PEG]Br_3_^−^ in ethanol.^a^

Entry	Substrate (**4**,**5**,**6**)	Product (**7**)	Time (h)	Yield (%)^b^

1	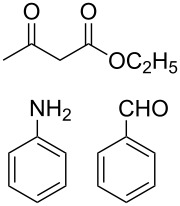	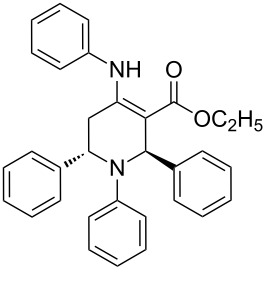	8	80
2	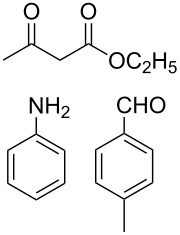	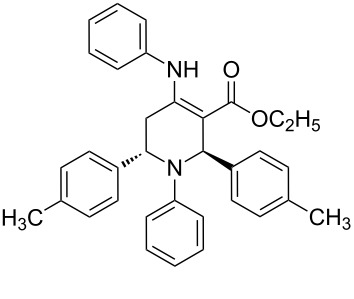	8	85
3	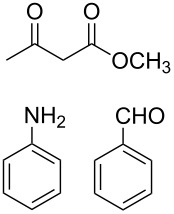	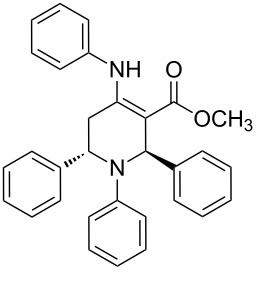	8	82
4	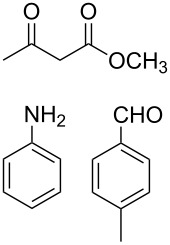	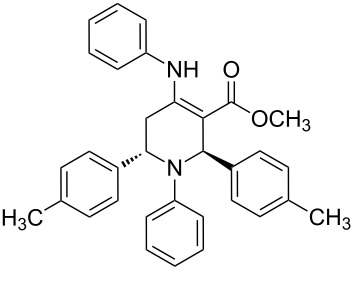	8	80
5	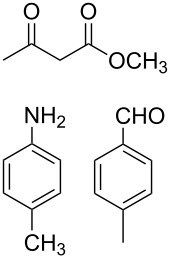	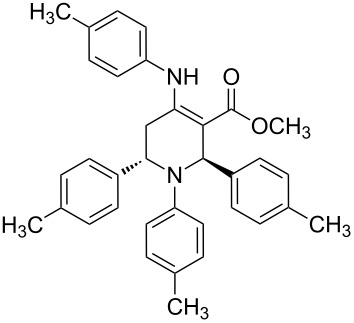	8	85
6	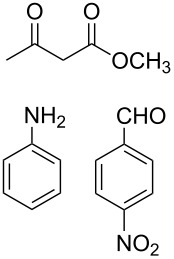	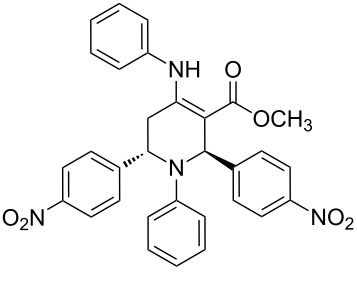	12	50
7	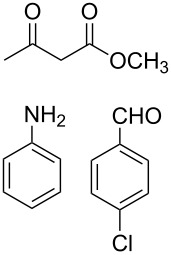	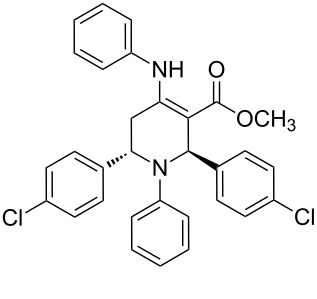	8	90
8	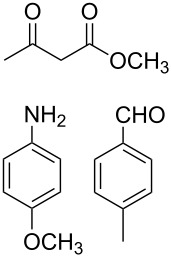	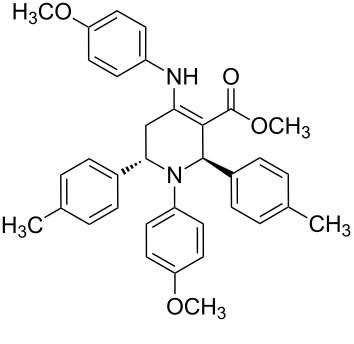	8	80
9	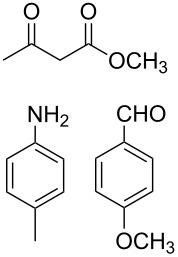	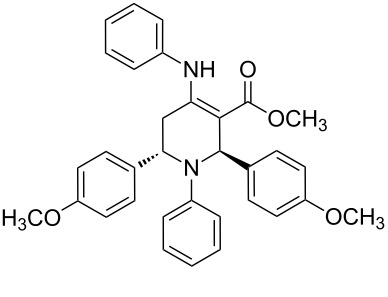	8	85
10	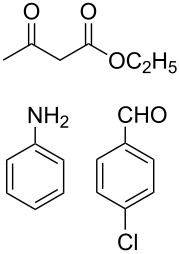	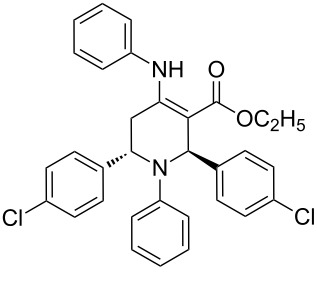	10	85
11	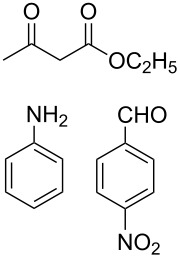	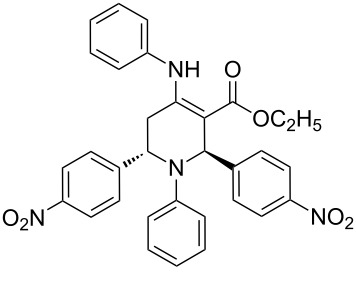	16	60
12	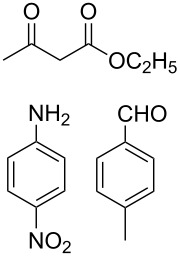	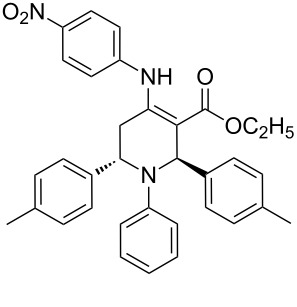	12	30
13	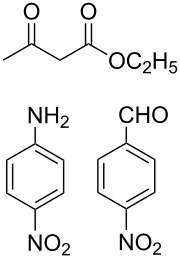	–	12	trace
14	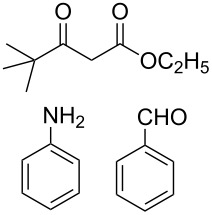	–	10	–

^a^Conditions: Aniline (2 mmol), benzaldehyde (2 mmol), β-keto ester (1 mmol), ethanol (2 ml) at room temperature. ^b^Isolated Yields.

Regeneration and recycling of the reagent **3** was checked through the reaction of aniline, benzaldehyde and ethyl acetoacetate as a model reaction. After completion of the reaction, the product was isolated by extraction with dichloromethane, and the resulting residue containing [K^+^PEG]Br^−^ was reused for the regeneration of the catalyst. The dropwise addition of bromine to the resulting mixture readily gave the PEG-wrapped KBr_3_ as a dark orange liquid, which was used for the subsequent run. The regeneration and recycling of the catalyst was checked for five runs. The catalytic activity remained almost unchanged for up to four runs (>78% yield), whereas it was found to decrease in further successive runs, and a very poor yield (about 45%) was obtained in the eighth run. In order to check the effect of the catalyst we carried out the coupling of aniline, ethyl acetoacetate and benzaldehyde without using any catalyst under the described reaction conditions. The reaction did not occur and unreacted substrates could be recovered even after a prolonged exposure time (12 h).

The synthesis of [K^+^PEG]Br_3_^−^ involves the reaction of [K^+^PEG]Br^−^ with liquid bromine involving the indirect use of toxic bromine, which makes this reagent undesirable from the point of view of sustainability. However, the inexpensive nature, easy availability of PEGs, higher stability, safer handling and efficient recycling of the catalyst make this more suitable and preferable than the existing organic ammonium tribromides [30–31].

**Scheme 3:**
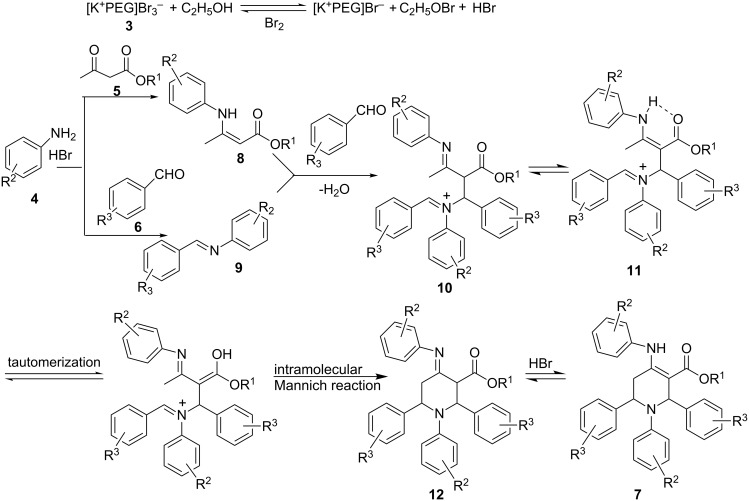
A plausible mechanism for the formation of piperidines.

The mechanism of this reaction is not clear at this stage, however, the reaction probably involves the in situ generation of HBr in the presence of ethanol, which could be the real catalyst for the present transformation. The probable mechanistic pathway is shown in Scheme 3, which is in analogy to the established mechanism as reported in the literature [30–31]. According to the proposed pathway, aniline reacts with β-ketoester and aldehyde in the presence of HBr to yield the corresponding enamine **8** and imine **9**, respectively. The subsequent attack of enamine on the activated imine, followed by inter- and intramolecular Mannich-type reactions, would yield the final piperidine derivative **7**. In order to support the proposed mechanism, we analyzed the reaction mixture by UV–vis spectroscopy at the end of the reaction; no bromine was detected in the reaction mixture, thus establishing the in situ generation of bromine from the reagent during the reaction.

## Conclusion

In summary, we have described the first time use of inexpensive, environmentally benign poly(ethylene)glycol to prepare an efficient and highly stable tribromide reagent through the concept of host–guest chemistry. The prepared PEG-embedded tribromide was found to be a highly efficient catalyst for the synthesis of highly substituted piperidines in the one-pot coupling reaction of aniline, aldehyde and β-keto ester. After completion of the reaction, the tribromide reagent can easily be regenerated by the addition of molecular bromine, which can be efficiently reused. Therefore the developed protocol not only involves the utilization of inexpensive PEGs but also its efficiency in recycling and its higher catalytic efficiency make it a more suitable and preferred catalyst over the existing bromine-based catalytic systems.

## Experimental

### Synthesis of [K^+^PEG]Br_3_^−^ (**3**)

Into the stirred mixture of equimolar amounts of PEG_400_ (10 mmol) and aq KBr (10 mmol), molecular bromine (10 mmol) was added dropwise under cooling conditions. The mixture was stirred for 4–5 h at room temperature. The resulting [K^+^PEG]Br_3_^−^, a dark orange-red viscous liquid, was dried under vacuum and used as the catalyst for the present reaction. The dark orange-red color of the catalyst disappeared during the reaction, and no bromine could be detected in the reaction mixture as analyzed by UV−vis spectroscopy; this established the in situ generation of bromine from the reagent during the reaction.

### Typical experimental procedure

Into a stirred mixture of aniline (0.2 g, 2.15 mmol), benzaldehyde (0.2 g, 1.89 mmol), ethyl acetoacetate (0.13 g, 1 mmol) in ethanol (2 ml) was added [K^+^PEG]Br_3_^−^ (**3**, 10 mol %). The reaction was continued for the time as presented in Table 1. The progress of the reaction was monitored by TLC. After completion of the reaction, the reaction mixture was concentrated under reduced pressure. The obtained residue was dissolved in dichloromethane to isolate the reaction product. The remaining layer containing [K^+^PEG]Br^−^ **2** was diluted with water and treated with bromine to regenerate **3**. The crude product was purified by column chromatography with ethyl acetate:hexane (4:6). The identity of the product was confirmed by comparing the physical and spectral analysis data with the reported compound [31].
